# Three-Dimensional Genome Architecture Influences Partner Selection for Chromosomal Translocations in Human Disease

**DOI:** 10.1371/journal.pone.0044196

**Published:** 2012-09-28

**Authors:** Jesse M. Engreitz, Vineeta Agarwala, Leonid A. Mirny

**Affiliations:** 1 Harvard-MIT Division of Health Sciences and Technology, MIT, Cambridge, Massachusetts, United States of America; 2 Broad Institute, Cambridge, Massachusetts, United States of America; 3 Biophysics Program, Graduate School of Arts and Sciences, Harvard University, Cambridge, Massachusetts, United States of America; 4 Department of Physics, MIT, Cambridge, Massachusetts, United States of America; University of North Carolina, United States of America

## Abstract

Chromosomal translocations are frequent features of cancer genomes that contribute to disease progression. These rearrangements result from formation and illegitimate repair of DNA double-strand breaks (DSBs), a process that requires spatial colocalization of chromosomal breakpoints. The “contact first” hypothesis suggests that translocation partners colocalize in the nuclei of normal cells, prior to rearrangement. It is unclear, however, the extent to which spatial interactions based on three-dimensional genome architecture contribute to chromosomal rearrangements in human disease. Here we intersect Hi-C maps of three-dimensional chromosome conformation with collections of 1,533 chromosomal translocations from cancer and germline genomes. We show that many translocation-prone pairs of regions genome-wide, including the cancer translocation partners *BCR-ABL* and *MYC-IGH*, display elevated Hi-C contact frequencies in normal human cells. Considering tissue specificity, we find that translocation breakpoints reported in human hematologic malignancies have higher Hi-C contact frequencies in lymphoid cells than those reported in sarcomas and epithelial tumors. However, translocations from multiple tissue types show significant correlation with Hi-C contact frequencies, suggesting that both tissue-specific and universal features of chromatin structure contribute to chromosomal alterations. Our results demonstrate that three-dimensional genome architecture shapes the landscape of rearrangements directly observed in human disease and establish Hi-C as a key method for dissecting these effects.

## Introduction

Chromosomal translocations affect cellular function by changing gene copy number, creating fusion genes with aberrant function, or repositioning regulatory elements. Classic examples of recurrent genomic rearrangements in cancer are the *BCR*-*ABL* translocation (observed in >90% of cases of chronic myeloid leukemia) and the *MYC-IGH* fusion (observed in ∼90% of cases of Burkitt's lymphoma) [1]–[4]. While these alterations play important roles in driving tumorigenesis [5] and directing targeted therapy in cancer patients [6], the factors that contribute to the formation of the thousands of translocations observed in human disease are not fully understood [7].

Repeated observation of specific translocations, as well as the existence of rearrangement hotspots in cancer [8], suggests that intrinsic cellular and genomic features predispose certain regions to translocate. Since fusion of two DSBs requires spatial contact, one attractive hypothesis is that higher-order genome organization – that is, the physical proximity of chromosomes in the nucleus *prior* to translocation – contributes to the occurrence of specific translocations [9], [10]. Indeed, work over the last decade has used fluorescence *in situ* hybridization (FISH) to show that the genes involved in several recurrent translocations are positioned relatively close to one another in the nuclei of normal cells [9]–[14]. However, current imaging methods lack the throughput needed to determine whether this phenomenon is broadly applicable, beyond anecdotal examples, to the thousands of translocations observed in cancer. In addition, the size of genomic regions involved in these interactions, as well as their tissue-specificity, remains uncharacterized.

While answers to these questions on a genome scale were previously unattainable, Dekker and colleagues recently developed a next-generation sequencing-based method, Hi-C, to describe contact probabilities across the entire human genome [15], providing a powerful new tool to investigate the relationship between 3D chromatin structure and translocation partner preferences. The combination of Hi-C and high-throughput translocation sequencing in a mouse pro-B cell line revealed that, given a uniform distribution of DSBs induced by random mutagenesis, frequencies of translocations between chromosomes are correlated with Hi-C contact probability prior to selection [16]. This result provided the first genome-wide demonstration that spatial proximity of loci influences patterns of translocations. However, the contributions of genome architecture to translocations observed in primary disease, as opposed to translocations induced experimentally, remains unclear.

To address this question, we leverage Hi-C to systematically test the hypothesis that human disease translocations occur between spatially proximal regions of the genome, integrating a total of 1,533 chromosomal rearrangements from both cytogenetic and sequencing-derived datasets. We find that many translocation partners are located in broad chromatin domains that are spatially proximal in normal cells, thus predisposing them to chromosomal rearrangements. Hi-C also identifies existing rearrangements in malignant cells and enables fine-mapping of chromosomal breakpoints. Our results support a broad role for three-dimensional genome structure in translocation-partner selection and establish Hi-C as a key method for dissecting the structural features of the genome that contribute to human disease.

## Results

### Strategy

**Figure 1:**
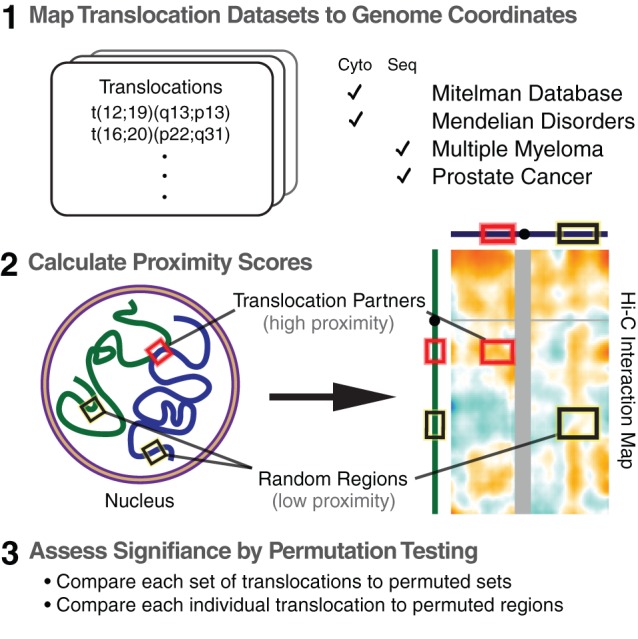
Schematic diagram of our approach.

Although previous studies have demonstrated that genome organization influences translocation partner selection, we wondered whether genome architecture contributes to rearrangements observed in human disease. To address this question systematically and on a genome-wide basis, we set out to test large human translocation datasets for evidence of proximity-mediated contact in genome-wide interaction maps for GM06990 lymphoblastoid and K562 erythroleukemic cell lines (Figure 1) [15]. Previous iterations of chromosome conformation capture technology have succeeded in identifying proximities between genes and regulatory elements [17], [18] as well as between actively transcribed or repressed genes [19], [20]. It was unclear, however, whether Hi-C could detect spatial proximities between translocation-prone loci (henceforth *translocation partners*). Thus we began by analyzing Hi-C data at translocation loci whose spatial relationships have been previously established by FISH.

### Validating Hi-C as a method for detecting proximity of translocation partners

**Figure 2:**
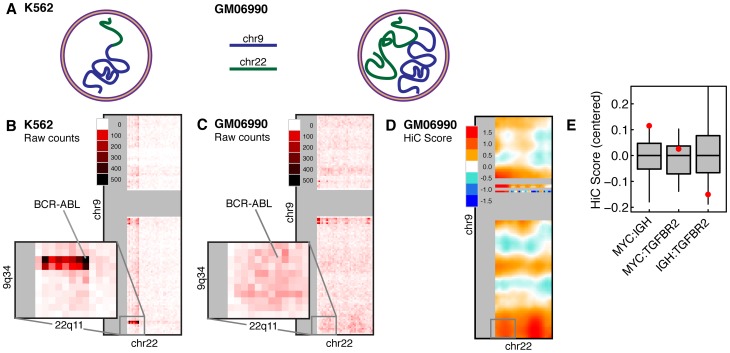
Hi-C detects interaction between known translocation partners *BCR*-*ABL* and *MYC*-*IGH.* (**A**) Chromosomes 9 and 22 are physically joined in K562 (left), but not in GM06990 (right). (**B**) Hi-C contact map of chromosomes 9 and 22 in K562 cells. Inset shows the chromosomal bands containing *BCR* (22q11) and *ABL* (9q34). High read counts identify the site of the *BCR-ABL* translocation. (**C**) Hi-C contact maps of chromosomes 9 and 22 in GM06990 cells, which show a relatively low read count in the *BCR-ABL* megabase bin compared to K562. (**D**) Normalized Hi-C contact map for chromosomes 9 and 22 in GM06990 shows elevated signal in the chromosomal bands containing *BCR* and *ABL*. (**E**) Centered interaction scores for the chromosomal bands containing the translocation partners *MYC-IGH* as well as the control partners *MYC-TGFBR2* and *IGH-TGFBR2*, compared to the background distribution of scores on the same chromosome pairs. Error bars represent the 5^th^ and 95^th^ percentiles.

Generated through a combination of proximity-mediated ligation and high-throughput sequencing, Hi-C interaction maps provide two-dimensional matrices of contact scores between megabase-sized regions on chromosome pairs. To verify that Hi-C faithfully detects proximity of known translocation loci, we first examined the canonical translocation partners *BCR* and *ABL*, which form an unbalanced, often-amplified rearrangement in the K562 human cancer cell line (Figure 2A) and have been shown by FISH to colocalize in the nuclei of multiple normal hematopoietic cell types prior to the translocation event [9], [21]. In K562 cells, where the *BCR-ABL* translocation has already occurred, Hi-C detected strong signal (1,996 reads) in the 1-Mb bin containing *BCR-ABL* (Figure 2B). We used Hi-C data to refine the likely breakpoint interval to the 50-kb regions chr9:132,550,000–132,600,000 and chr22:21,950,000–22,000,000, consistent with prior breakpoint identification for the BCR-ABL translocation [22]. Interestingly, the raw observed Hi-C counts showed a characteristic pattern for an unbalanced translocation, with peak signal at a corner decaying in a single direction along both chromosomes (Text S1, Figure S1). This signature was also present at two other genomic loci at which translocations have been previously reported in cytogenetic studies of the K562 cell line; Hi-C data enabled the fine-mapping of the likely breakpoint regions for these potentially functional rearrangements for the first time (Text S1, Figure S1, Figure S2, Table S1).

Having demonstrated that the Hi-C data provides robust evidence for translocations that have already occurred, we asked whether there would be evidence for spatial proximity of *BCR* and *ABL* in cells *prior* to translocation. In immortalized, karyotypically normal lymphoblastoid cells (GM06990) that do not harbor the translocation (Figure 2A), the signal in the megabase-bin containing *BCR*-*ABL* was markedly reduced (10 reads, Figure 2C) as compared to the signal seen in K562 cells. We expected this large difference in raw read counts between the two cell lines because these regions of chromosomes 9 and 22 interact in *cis* in K562 and *trans* in GM06990.

While the raw read data hinted at an enrichment of contact frequency between the *BCR* and *ABL* loci (Figure 2C), the read count observed in a 1-Mb bin in Hi-C is affected by confounding factors such as GC content, abundance of restriction enzyme sites, and sequence mappability [15], [23]. To account for these biases in Hi-C data, we applied the normalization scheme developed by Yaffe and Tanay [23]. In the rest of this work, we represent proximities using the *Hi-C Score,* defined as the *log2* enrichment of observed reads to expected reads using a probabilistic correction model as described [23]. We note, however, that we obtained similar results with the original approach to Hi-C normalization described by Dekker and colleagues (data not shown) [15].

Using a normalized contact map of Hi-C scores, we again examined *BCR-ABL* in GM06990 cells (Figure 2D). In the karyotypically normal GM06990 cell line, the megabase bins containing *BCR* and *ABL* displayed higher proximity compared to other sites on the same chromosomes. Furthermore, the region of high proximity extended to include much of 9q34 and 22q11. Compared to random regions of the same size on chromosomes 9 and 22, the chromosomal bands containing *BCR* and *ABL* fell above the 95th percentile of proximity scores in GM06990 cells. This suggested that Hi-C detected significant contact frequencies between the translocation-prone *BCR* and *ABL* loci in normal cells above the background distribution of Hi-C scores between loci on chromosomes 9 and 22.

As an additional validation that Hi-C could accurately detect the spatial proximity of translocation-prone loci, we examined the loci involved in the t(8;14)(q24;q32) translocation, a rearrangement associated with Burkitt's lymphoma that brings the oncogene *MYC* under the control of activating enhancer elements at the *IGH* locus. Osborne and colleagues demonstrated by FISH that induction of B lymphocytes causes relocation of *Myc* and *Igh* to common transcription factories, bringing these translocating regions into close proximity [24]. In Hi-C data derived from lymphoblastoid cells, the chromosomal bands including *MYC* and *IGH* showed higher contact frequency than other regions of the same size on the same chromosome pair, thus representing a local hotspot of proximity between chromosomes 8 and 14 (Figure 2E). In comparison, the proximity scores for the control loci *MYC-TGFBR2* and *IGH-TGFBR2*, which are not observed to translocate in cancer, did not differ significantly from background. Notably, these results exactly paralleled the FISH results obtained for the same loci in a similar cell type [12]. We conclude that Hi-C can specifically detect the proximity of translocation partners in karyotypically normal cells.

### Many translocation partners are spatially proximal in the nucleus

Having found that Hi-C is suitable for examination of proximity-mediated interactions between translocation-prone loci, we then proceeded to test the main hypothesis of this study: that spatial proximity influences the formation of many translocations observed in human disease genome-wide. First, we gathered four datasets totaling 1,533 human chromosomal rearrangements (Table 1). Identified by cytogenetic and high-throughput sequencing modalities in cancer and germline genomes from multiple tissues, these four genome-wide datasets broadly sampled the space of chromosomal translocations observed in human disease (Methods). Importantly, the Mitelman Database and multiple myeloma datasets contained hundreds of inter-chromosomal translocations from lymphoid-derived malignancies, matching the cell lineage of our lymphoblastoid Hi-C data. For each human disease translocation, we mapped the chromosomal bands implicated (typically representing multi-megabase-sized regions) to genomic coordinates, and then calculated average Hi-C contact frequencies across each pair of partner regions. To statistically evaluate sets of translocations for evidence of increased spatial proximity, we devised a permutation-based approach that corrected for potential biases including 1) systematic differences in association between pairs of chromosomes, 2) regions of the genome that have strong proximity signal with many other regions, 3) sizes and positional biases of regions in our translocation sets, and 4) broad chromatin features (Figure 1, Methods).

**Table 1:** Translocation datasets and permutation results.

Dataset	Mean Hi-C Score (Translocations)*	Mean Hi-C Score (Permutations)*	Permutation P-Value	Rank-Sum P-Value	Individual Significant Translocations	Total Unique Translocations	% Genome Covered	% Interactions Covered**	Source
Mitelman Database	0.17±0.03	0.052±0.001	<0.001	6.42E-23	7	577	79.7%	1.12%	Mitelman et al. 2011
*Blood*	0.19±0.03	0.063±0.001	<0.001	2.13E-20	6	440	72.4%	0.87%	
*Non-Blood*	0.10±0.05	0.018±0.001	<0.001	1.78E-04	1	137	45.2%	0.27%	
Multiple Myeloma	0.17±0.09	0.039±0.002	<0.001	7.27E-03	2	89	22.9%	0.03%	Chapman et al. 2011
Prostate Cancer	0.07±0.04	0.002±0.001	<0.001	7.92E-04	0	89	21.9%	0.03%	Berger et al. 2011
Mendelian	0.04±0.02	0.006±0.001	<0.001	4.05E-03	1	779	91.5%	0.79%	https://www1.hgu.mrc.ac.uk/Softdata/Translocation/

* Bounds represent a 95% confidence interval.

** Percentage of inter-chromosomal 1-Mb bins that are covered by translocations.

**Figure 3:**
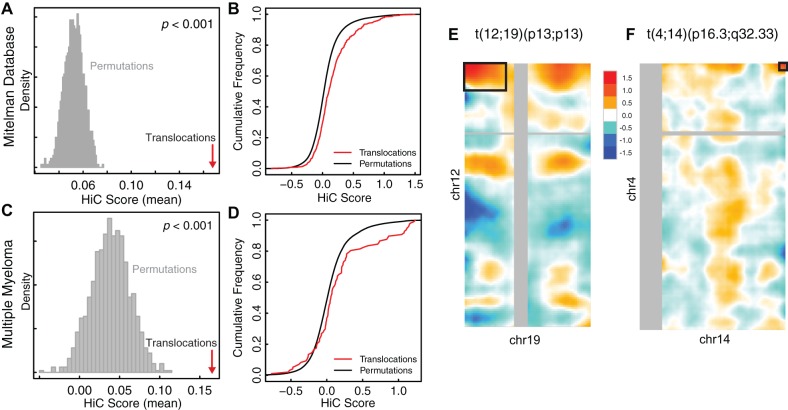
Many translocation partners exhibit high contact frequencies in the normal nucleus. Permutation test results for blood translocations from the Mitelman (**A and B**, *n* = 577) and multiple myeloma datasets (**C and D**, *n* = 89). Histograms (**A and C**) represent the distribution of the mean proximity score within each of 1,000 permuted sets of *n* translocations that preserve the characteristics of the true set (Permutation Method 1, see Methods). Red arrow indicates the mean of the proximity scores for *n* real translocations. Cumulative distribution plots (**B and D**) compare the distribution of the *n* scores for real translocations versus the distribution of the 1000*n* scores for permuted translocations. Heatmaps show Hi-C contact maps in GM06990 cells for two significant individual translocations: t(12;19)(q13;p13) (**E**) in the Mitelman Database and t(4;14)(p16.3;q32.33) (**F**) in the multiple myeloma dataset. Black boxes indicate the chromosomal bands containing the translocation breakpoints.

In all four translocation sets tested (Mitelman recurrent cancer, multiple myeloma, prostate cancer, and rare Mendelian translocations), we found that translocation partners have higher Hi-C contact frequencies than permuted regions with similar characteristics (Table 1, Figure S3). This signal was present in Hi-C contact maps from the karyotypically-normal GM06990 lymphoblastoid cells, suggesting that spatial proximity of disease translocation partners precedes the rearrangement. Although the magnitude of the effect was incremental compared to the overall distribution of *trans* Hi-C scores (Figure S4), the finding was statistically significant (*P*<0.001, permutation test) in all four datasets (Table 1; Figure 3A, C). A closer examination of the distribution of proximity scores for true and permuted translocations showed that this signal arose from the sum of small effects across a broad range of translocations, rather than from large effects from a smaller number of rearrangements (Figure 3B, D; Figure S3). When we performed the same analysis using the Hi-C contact map generated with a different restriction enzyme in the same cell line, we observed nearly identical results (Table S2).

In addition to examining sets of translocations, we tested individual translocation partners for increased spatial proximity by generating permuted regions for each translocation. Consistent with the observation that the significant group signal was due to a sum of small effects, relatively few translocations showed individually significant contact frequencies (Table S3). For those translocations that were individually significant, spatial proximity may play a particularly important role in their formation. Two of the high-scoring translocations included 19p13, the site of the *TCF3* gene, which is frequently found translocated in acute lymphoblastic leukemia (Figure 3E) [25]. One individually significant translocation involved the chromosomal band containing the *IGH* locus: t(4;14)(p16;q32) is frequently found in multiple myeloma patients, causing dysregulated expression of *FGFR3* and/or *WHSC1* (Figure 3F) [26], [27]. Our results suggest that for these individual translocations, spatial proximity prior to translocation may be especially important. These may be strong candidate translocations for experimental investigation of this phenomenon.

Our analysis supports a model where translocation partners reside in broad interacting domains that span multi-megabase chromosomal regions (Figure 3E, F). These broad interactions, occurring across an aggregate cell population, bring translocation partners into close spatial proximity, increasing the likelihood of rearrangement. Still, we wondered whether Hi-C might detect significant smaller-scale proximities, on the scale of a megabase. We mapped translocations from the whole-genome sequencing studies to megabase-sized bins and reran our four permutation tests on this dataset, comparing each translocation to other megabase-sized pairs of regions. We found that the one-megabase-bins containing translocation breakpoints were more spatially proximal than expected by chance in both multiple myeloma and prostate cancer (Figure S3). While suggestive, we cannot definitively conclude that the 1-Mb bins containing the translocations are more frequently in contact than the broader chromosomal bands containing them due to the sparse read-density at this scale.

### Tissue-specific effects

Multiple lines of evidence suggest that genome organization is tissue-specific and context-dependent. Gene-level or chromosomal contacts exhibit reproducible changes across tissue types, time points [21], [28], [29], and perturbations [14], [30]. We therefore hypothesized that evidence for spatial proximity in Hi-C data from a lymphoblastoid cell line would be highest for translocation partners observed in hematologic malignancies. Indeed, we found that recurrent translocations observed in blood cancers in the Mitelman database displayed higher Hi-C contact frequencies than translocations observed in non-blood tumors (*P* = 2×10^−3^, Wilcoxon rank-sum test). For example, the translocation partners for t(12;15)(p13;q15), a rearrangement found in acute lymphoblastic leukemia and lymphoblastic lymphoma, showed much more significant proximity (*P*<0.05, permutation test) in GM06990 cells than another pair of translocating loci on the same chromosomes, t(12;15)(p13;q26), found in fibrosarcoma (*P* = 0.28, permutation test). These results suggest that tissue-specific changes in genome organization may predispose specific regions to translocate in different malignancies. At the same time, however, the significantly elevated Hi-C contact frequencies between translocation partners in non-matching cell types implies the presence of universal features of spatial genome organization. Additional Hi-C experiments in multiple matched cell types may help to elucidate lineage-dependent variation in global chromosomal conformation and its contribution to translocation partner selection.

### Genomic features of translocation breakpoints

**Figure 4:**
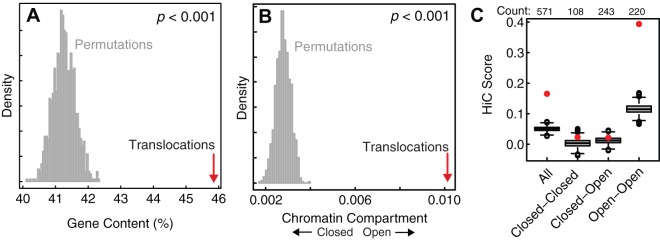
Features of translocation breakpoints. Comparison of average (**A**) gene content (% bases spanned by transcripts; includes both exons and introns) and (**B**) chromatin compartment score for Mitelman database translocations (red) and 1,000 permuted sets, each of size *n* = 571, that preserve the characteristics of the true set. Chromatin compartment scores are calculated using the first eigenvector of the Tanay-normalized correlation matrix (see Methods). Positive and negative scores indicate open and closed chromatin compartments, respectively. (**C**) Mean Hi-C interaction scores for Mitelman blood translocations (red dots) compared to sets of permuted regions selected from the same chromatin compartments (gray boxplots).

One explanation for the observed spatial proximity between translocation partners is that these regions have preferentially high gene content, or lie in the open chromatin compartment, rendering them easily accessible and mutable. We tested this and found that translocation breakpoints were significantly enriched for gene-rich, euchromatic regions in all four datasets (Figure 4A, B), consistent with the biased occurrence of DSBs in transcriptionally active chromatin [31], [32]. To determine whether translocations partners have higher contact frequencies compared to other regions of similar chromatin state, we repeated our permutation tests controlling for chromatin compartment (open versus closed, see Methods). We found that translocation partners still are more spatially proximal than expected by chance, although the significance of this finding was reduced across all datasets (Table S4). Translocations whose partners resided in the open chromatin compartment were more significantly spatially proximal than translocations with one or both partners in the closed compartment (Figure 4C). The increased contact frequencies and DSB occurrences in transcriptionally active chromatin may contribute to the observation that tissue-matched translocations correlate more closely with Hi-C signal.

## Discussion

In this work, we provide evidence that many translocation breakpoints observed in human diseases have high Hi-C contact frequencies in normal cells, suggesting a broad role for 3D chromatin organization in determining the frequency of translocations between partner loci. Previous data about this phenomenon is derived primarily from FISH [9], [12]–[14], [33], a technique that, although revealing, can investigate only a limited number of loci simultaneously. Chromosome conformation capture with Hi-C, in contrast, allows genome-wide, unbiased investigation of proximity-mediated interactions between translocation partners [16]. Since Hi-C measures contact frequencies rather than average nuclear distances, this approach provides a test for the “contact first” model of genomic rearrangements.

We find that Hi-C detects frequent proximity of hundreds of translocation-prone loci, including 1) translocations recurrently observed by cytogenetics in primary cancers from multiple tissues, 2) unbiased collections of rearrangements detected in tumors by next-generation sequencing, and 3) translocations associated with rare Mendelian disorders. In all cases, we detect a subtle but significant enrichment for translocation-partner contacts when compared to a null distribution. Recurrent translocations from a matched cell lineage (hematopoietic malignancies) show a stronger Hi-C contact signal than translocations from tumors derived from other lineages, suggesting that tissue-specific chromosome conformation may contribute to rearrangement partner selection. We also identify several individual translocations that show particularly strong Hi-C contact frequencies, including t(12;19)(p13;p13) and t(4;14)(p16.3;q32.33), which are both recurrently found in multiple hematopoietic tumor types. We thus predict that these individual translocations, particularly those that do not involve the *IGH* locus (see below), have a particularly high probability of rearrangement due to their increased frequency of contact.

Although translocations often occur in areas of high Hi-C contact frequency, many translocations occur in areas of low or average Hi-C contact frequency. This may occur for several reasons. First, it is possible that even though the “contact first” model is responsible for the occurrence of some translocations, this signal cannot be detected in Hi-C data from a population of cells. Spatial genome organization may be unique to individual cells or sub-populations of cells, and thus aggregate Hi-C signal may fail to capture spatial interaction occurring in only a few cells (and it may be these cells that go on to form translocations under strong positive selection in the cancer).

Second, translocations that do not show an elevated Hi-C signal in our analysis may have occurred for reasons entirely unrelated to spatial co-localization. While we directly test the “contact first” model, spatial proximity is certainly not a sufficient condition for observing a translocation in disease genomes [7]. Some translocations may occur under a “breakage-first” model, whereby cellular mechanisms exist to co-localize DSB ends following breakage [34]. The frequency of observed translocation is also affected by multiple other factors, potentially including DSB susceptibility [16], DSB mobility [34], spatial heterogeneity among cells, positive selection, and ascertainment bias. To dissect the contributions of other cellular processes to translocation partner selection, investigators will need to examine concurrent genome-wide profiles of these phenomena in the same cellular system.

It is also worth nothing that while we detect a significant enrichment of spatial proximity between observed translocation partners, the datasets analyzed here were not sufficient to directly test whether the formation frequencies of these translocations are correlated with the contact frequencies between genomic partners, as has been shown experimentally for induced translocations [16]. This is because the true frequency with which translocations *form* is quite different from the frequency with which they have been *observed* in clinical databases. Observed frequencies are biased by factors including positive selection during neoplastic progression, the limited sensitivity of current methods to detect rare rearrangements in clinical samples, and ascertainment biases intrinsic to non-whole genome testing and clinical sample collection. While we do, despite these factors, observe significant correlation between the number of reports of a translocation in the Mitelman database and the Hi-C interaction score (Figure S5), we do not detect significant correlation (*T*-test *p*-value  = 0.532) between the number of observations of a translocation and the Hi-C interaction permutation *p*-value (the more robust measure of spatial proximity).

In lymphoid malignancies, the spectrum of observed translocations is drastically altered by the presence of the DSB-inducing enzyme AID, which contributes to the formation of rearrangements involving loci throughout the genome, particularly the Ig locus [31], [32]. The formation of DSBs in these regions may then be a dominant force in determining which loci rearrange. Our results suggest that proximity plays a role in the formation of translocations in other tumor types as well. Indeed, given the absence of AID in non-lymphoid tissues, proximity might play a relatively larger role in determining the landscape of observed rearrangements in non-lymphoid cancers. Thus we suggest that future investigations of spatial proximity in cancer will benefit from examination of chromatin architecture in non-lymphoid tissues.

In an important methodological demonstration, we show the utility of Hi-C data in the discovery and fine-mapping of existing translocations in malignant cells genome-wide (Figure 2B, Figure S1), in accord with previous work using the targeted 4C method [35]. This finding has two key implications for future genome-wide analyses of chromatin structure in cancer cells. First, Hi-C data is able to accurately detect translocation breakpoints, allowing genome-wide analysis of structural rearrangements. Indeed, Hi-C data may be able to reconstruct the complete karyotype of a cancer cell, including deletions, amplifications, inversions, and other chromosomal alterations. Second, this analysis will be critical in filtering out the effects of chromosomal translocations that might interfere with the study of other *trans* proximity-mediated interactions in future Hi-C studies.

Given the role of spatial proximity in translocation partner selection demonstrated in this study, the molecular mechanisms that govern three-dimensional genomic architecture in normal and cancerous cells may prove important in our understanding of cancer etiology. Work to characterize the interactions between chromosome conformation and triggers for rearrangements will help to untangle the molecular processes of damage and aberrant repair that contribute to oncogenic transformation.

## Methods

### Hi-C chromosomal conformation capture

We used public Hi-C data (GEO accession GSE18199) generated to interrogate the long-range genomic interactions in the GM06990 lymphoblastoid and K562 erythroleukemic cell lines [15]. For raw count data, we used the mapped reads and the one-megabase binning scheme for the data generated using HindIII as described [15]. To control for differences in coverage, location of restriction enzyme recognition sites, mappability, and other features unique to each one-megabase bin, we obtained the normalized contact maps generated by Yaffe and Tanay [23]. Briefly, this normalization method learns a probabilistic correction model based on uniquely-mapped reads in each Hi-C dataset, then applies a linear smoothing filter to calculate the number of reads expected in each 1-Mb bin. When compared to previous normalization approaches, this method significantly improves the correlation of the 1-Mb genome-wide contact maps generated with different restriction enzymes. We call the *log* ratio of observed to expected read counts under this model the *Hi-C Score*. We applied the *log* variance-stabilizing transformation to reduce the contributions of strong outliers when calculating summary statistics over a region.

### Translocation datasets

We collected four large inter-chromosomal translocation datasets, derived both from karyotyping and high-throughput sequencing studies. First, we collected a set of recurrent *trans*-chromosomal cancer translocations that have been observed in multiple patient cases [36]. The Mitelman Database describes translocations using chromosomal bands; precise breakpoints were not available. The average size of defined chromosomal translocation bands in this database was large (∼10 Mb). While positive selection modifies the frequency of cancer translocations, particularly driver rearrangements, we expected that many of these recurrent translocations were predisposed to recur due to factors such as genome organization.

Translocations from multiple myeloma and prostate cancer were identified from whole-genome or exome sequences using the *dRanger* algorithm [37], [38]. We used translocations with at least three supporting reads in our analysis. Compared to the Mitelman Database, we expected catalogs of translocations in primary tumors to contain a higher frequency of passenger rearrangements, as well as a higher proportion of private mutations that occurred stochastically rather than systematically due to predisposing factors.

Finally, we collected all two-partner inter-chromosomal translocations (*n* = 947) associated with Mendelian syndromes from the Disease Associated Chromosomal Rearrangement Database (https://www1.hgu.mrc.ac.uk/Softdata/Translocation/). Again, precise breakpoints were not available for this dataset. Because these translocations can cause severe phenotypes, many of these mutations are not transmitted through generations: these diseases, though rare, are caused by recurrent *de novo* translocations. In addition, these genomic rearrangements do not experience the same positive selective pressures as the cancer translocations, complementing the previous datasets.

For the first Mitelman and Mendelian translocation databases, we mapped the cytogenetic bands (e.g. t(9;22)(p13;q13)) to human genome coordinates using the UCSC Genome Browser Build hg18. We considered 3-way translocations as 3 distinct two-way translocations, and excluded all translocations involving more than 3 partners. We also excluded translocations involving entire chromosomal arms, and did not include any intra-chromosomal rearrangements (*e.g.*, duplications or inversions).

### Chromatin compartment and gene content

We assigned regions to chromatin compartments using principal component analysis on the Tanay-normalized contact map as described [15]. Positive and negative scores indicate open and closed chromatin compartments, respectively, and correlate with other genomic features such as gene content, histone modification, and DNase I hypersensitivity. For each translocation region, we calculated a compartment score as the mean of the principal component values for all overlapping megabase bins. We represented gene content as the percentage of bases covered by RefSeq genes, including both exons and introns.

### Permutation testing

We employed a permutation strategy to search Hi-C data for evidence of proximity between translocation breakpoints. We calculated the *proximity score* for each translocation region as the mean normalized Hi-C score of all 1-Mb bins overlapping the chromosomal band involved in the translocation. When calculating these summary statistics, we did not include bins that 1) overlapped centromeres or 2) had no coverage across the entire dataset. To assess the significance of individual translocations, we generated a null distribution by considering 1,000 random pairs of regions with one of four permutation methods:

1. We selected regions of identical size from the same chromosome pair. This within-chromosome permutation scheme controlled for the systematic differences in association between pairs of chromosomes: smaller, gene-rich chromosomes, for instance, tend to group together [15].
2. We fixed one region, and selected as a partner a random region of identical size on the same chromosome. This controlled for features of the translocation partners that might predispose them to interact with many other regions on the same chromosome.
3. We fixed one region, and selected as a partner a random region of identical size on any other chromosome. This controlled for features of the translocation partners that might predispose them to interact with many other regions across the genome.
4. We fixed one region, and selected as a partner a random region from the entire set of translocations partners that did not fall on the same chromosome as the fixed partner.

We observed similar results for all four permutation methods (Table S4); we present results from Permutation Method 1 in the main text. In all cases where we selected random regions, we required that less than 50% of the bins in the random region overlapped with centromeric regions or bins with no coverage across the entire dataset.

For each individual translocation, we calculated the *p*-value for each translocation as the fraction of permuted locations that exceeded its proximity score, and corrected for multiple hypothesis testing using the Benjamini-Hochberg method. We assessed the significance of each translocation dataset as a group using a similar approach. For each of our four datasets, we generated 1,000 randomized datasets that preserved the overall properties of the group of translocations: the chromosome pairings and region sizes matched the original set. We calculated a summary score for each of these randomized datasets that represented the mean interaction score across all translocations, and calculated a *p*-value by comparing these statistics to the null distributions. We also assessed the differences between the proximity scores of the true and randomized data distributions using the two-sided Student's *t* test and Wilcoxon rank sum test to monitor the degree to which outliers drove the result in the permutation scheme.

### Permutations within chromatin compartment

We also evaluated the significance of our results by controlling for chromatin compartment in Permutation Methods 1–3. To accomplish this, we allowed swapping only within compartments. For each translocation partner, we calculated the chromatin score and chosen randomly from similarly-sized regions whose chromatin scores had the same sign.

### Fine mapping

To identify likely chromosomal breakpoints responsible for previously reported translocations, we first identified the 1 Mb×1 Mb bin across the chromosome pair with the highest total normalized read count. We then selected all reads mapping to a 3 Mb×3 Mb window around this bin. We then counted the number of observed reads mapping to 50 Kb×50 Kb bins, and looked for a pattern characteristic of unbalanced translocation. We then selected the corner-most 50 Kb bin, and counted reads mapping to 1 Kb×1 Kb regions within this larger bin. In some cases, the read count was sufficient to allow breakpoint identification at this fine scale, but in other cases read coverage was too sparse to further localize the breakpoint. In all cases, resolution is limited by the density of HindIII restriction sites.

## Supporting Information

Figure S1 Fine mapping of previously-reported inter-chromosome translocations in K562. Heatmaps showing the observed number of reads mapping to 50 kb×50 kb bins at the *BCR*-*ABL* locus (**A**), the *CDC25A*-*GRID1* locus (**B**), the *NUP214*-*XKR3* locus (**C**), three sites of previously reported inter-chromosomal translocations in the K562 cell line.
(EPS)

Figure S2 Fine mapping of previously reported translocations in the K562 cell line. Heatmaps showing the observed number of reads mapping to 50 Kb bins at selected regions of (**A**) *BCR*-*ABL* and (**B**) the novel t(3;10) *CDC25A*-*GRID1* translocation. (**C**) Gene expression for dysregulated translocation partners (1), normally-regulated translocation partners (2), and constitutively-expressed myeloid genes (3) in MV4-11 (AML) and K562 (CML) cell lines. Expression values for each gene are normalized to the median expression for all genes. Note that *XKR3*, the translocation partner of *NUP214*, is not assayed on this microarray platform. (**D**) CML-to-AML log_2_ fold-change for all assayed genes, sorted in increasing order. Red lines indicate the fold-change for labeled genes. The dysregulated translocation partners *CDC25A*, *NUP214*, and *ABL1* are highly upregulated in CML, all falling in the upper quartile of genes in terms of fold-change. (**E**) ENCODE ChIP-seq data for transcription factors and H3K4me1 near the predicted *GRID1* breakpoint [39]. Color for H3K4me1 corresponds to cell type. Color for transcription factor data is proportional to the ChIP-seq signal. Data was viewed with the UCSC Genome Browser, genome build *hg18.*
(EPS)

Figure S3 Permutation test results for all databases. Histograms (gray) represent the mean proximity scores within each of 1,000 permuted sets of translocations (Permutation Method 1) that preserve the characteristics of the true set. Red line denotes the mean proximity score of the true translocation set. Cumulative frequency plots compare the score distributions for observed and permuted translocations.
(TIFF)

Figure S4 Distribution of Hi-C Scores for all *trans* bins. Histogram of Hi-C scores (*log_2_* observed/expected read counts) for all one-megabase *trans*-chromosomal bins in GM06990. Expected read counts are calculated on a per-bin basis to control for differences in coverage, mappability, and HindIII restriction sites (see Methods).
(TIFF)

Figure S5 Correlation between translocation frequency and contact frequency for Mitelman translocations. Boxplots of (A) permutation *p*-values and (**B**) normalized Hi-C scores for translocations binned by number of occurrences in the Mitelman database.
(EPS)

Text S1 Hi-C fine-mapping of translocations in the K562 cell line.
(PDF)

Table S1 Fine-mapping known K562 translocations.
(PDF)

Table S2 Individual translocation-prone loci that significantly colocalize in normal nuclei.
(PDF)

Table S3 Permutation results for NcoI Hi-C data.
(PDF)

Table S4 Permutation results after controlling for chromatin compartment (HindIII).
(PDF)
