# Supplementary figures and images for: Three-Dimensional Genome Architecture Influences Partner Selection for Chromosomal Translocations in Human Disease
Source: PLoS One. 2012 Sep 28;7(9):e44196. doi: 10.1371/journal.pone.0044196 (PMC3460994; doi:10.1371/journal.pone.0044196)

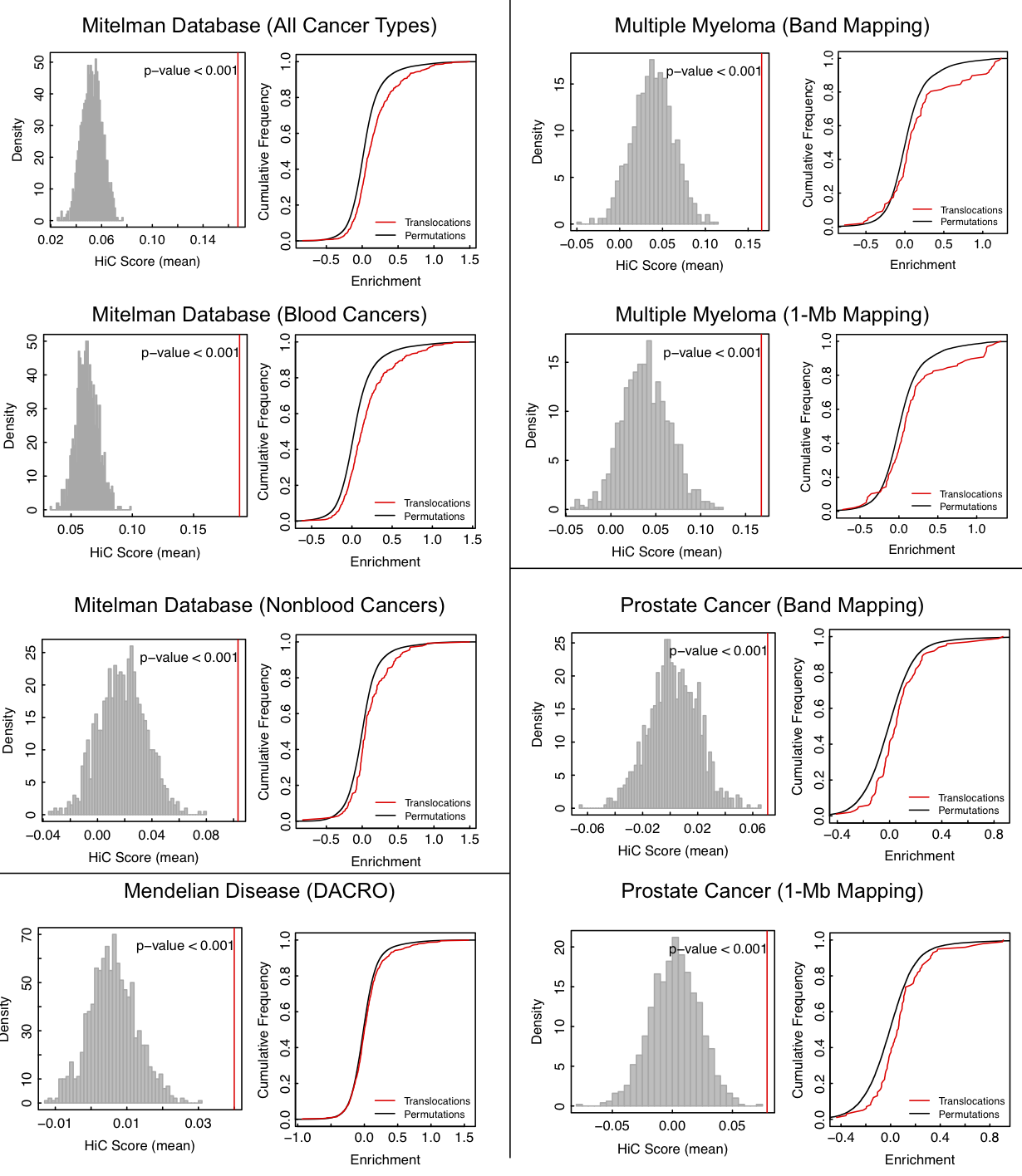

Supplement: Figure S3 — Permutation test results for all databases. Histograms (gray) represent the mean proximity scores within each of 1,000 permuted sets of translocations (Permutation Method 1) that preserve the characteristics of the true set. Red line denotes the mean proximity score of the true translocation set. Cumulative frequency plots compare the score distributions for observed and permuted translocations. (TIFF) [file pone.0044196.s003.tiff]

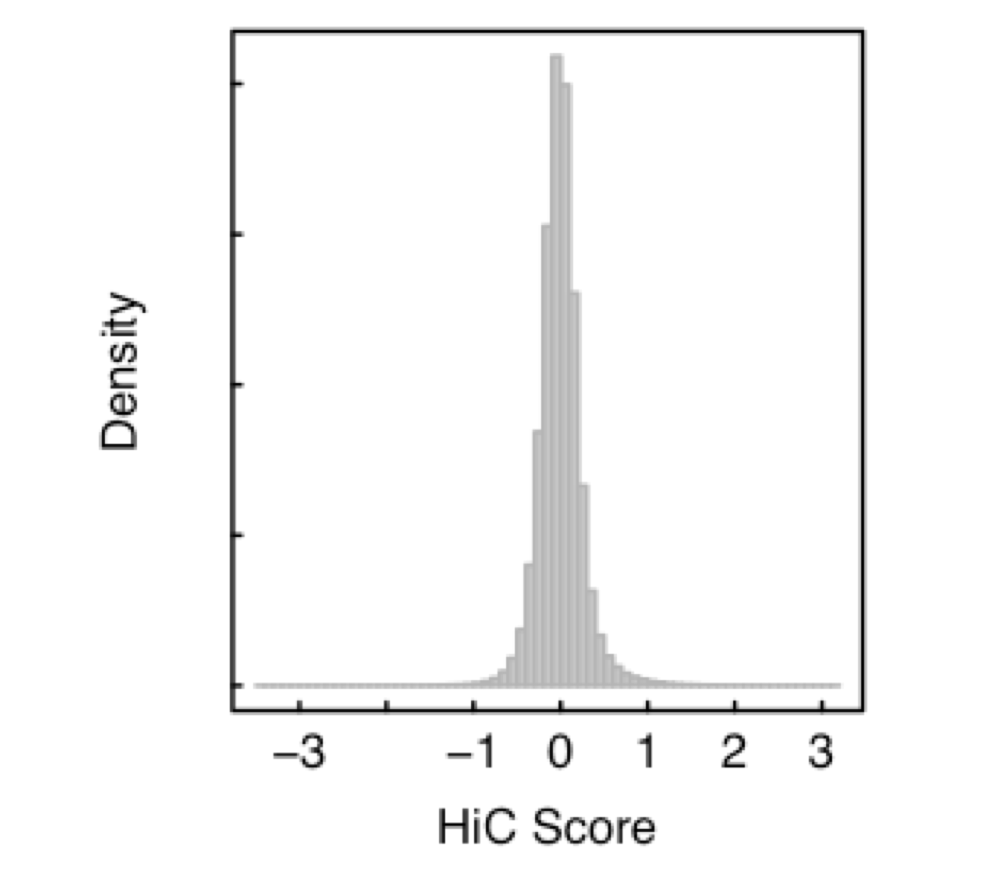

Supplement: Figure S4 — Distribution of Hi-C Scores for all trans bins. Histogram of Hi-C scores (log2 observed/expected read counts) for all one-megabase trans-chromosomal bins in GM06990. Expected read counts are calculated on a per-bin basis to control for differences in coverage, mappability, and HindIII restriction sites (see Methods). (TIFF) [file pone.0044196.s004.tiff]
