# Supplementary material for: Three-Dimensional Genome Architecture Influences Partner Selection for Chromosomal Translocations in Human Disease
Source: PLoS One. 2012 Sep 28;7(9):e44196. doi: 10.1371/journal.pone.0044196 (PMC3460994; doi:10.1371/journal.pone.0044196)
Supplement: Text S1 — Hi-C fine-mapping of translocations in the K562 cell line. (PDF) [file pone.0044196.s006.pdf]

Supporting Text

Fine-mapping of translocations in K562 cell line. Hi-C data provides evidence not only for pre-translocation chromosomal proximity, but also for translocations that have already occurred (Figure 1). We thus attempted to use Hi-C data to fine-map chromosomal breakpoints in K562 cells. Although K562 is a widely used cancer cell, the exact breakpoints and genes implicated in its translocations remain largely unknown. We assembled a set of 13 previously described, coarsely-mapped translocations in K562 cells (Table S1), and explored the raw Hi-C data at these loci to locally refine breakpoint intervals. We observed a local hotspot of inter-chromosomal reads for 6 of these 13 previously described rearrangements; signal was highest at the location of the chromosomal breakpoints, where ligation has occurred, and decayed with distance away from the breakpoints along the fused chromosomal bands. At 3 of the 6 regions, raw Hi-C read counts at 50-kb resolution showed a characteristic pattern for an unbalanced translocation, with peak signal at a corner decaying in a single direction along both chromosomes (Figure S1A-C).

At the 6 loci at which Hi-C signal was detected, we counted the number of raw Hi-C reads mapping to 50-kilobase intervals in the broad region of peak interaction signal. At 3 of the 6 regions, a heat map showing read counts at 50-kb resolution showed a pattern of peak signal at a corner, with signal decaying in a single direction along both chromosomes (Figure S1A-C). This is an expected pattern for an unbalanced translocation; signal is highest at the location of the chromosomal breakpoints, where ligation has occurred, and decays with distance away from the breakpoints along the fused chromosomal bands. For the *BCR-ABL* translocation, for example, we mapped the breakpoint to a 50-kb region spanning chr9:132,550,000-132,600,000 and chr22:21,950,000-22,000,000 (Figure S1A, Table S1). Within this region, three reads (the highest local density) mapped to within a kilobase of the described precise breakpoint for the *BCR-ABL* translocation in the K562 cell line (chr9:132,596,950-132,597,013 and chr22:21,962,697-21,962,754) [1].

We next attempted to identify the breakpoint region for the other loci showing Hi-C signal characteristic for a translocation in the K562 cell line. Sequence-based identification of fusion gene transcripts in the K562 cell line has recently identified a second translocation between chr9 and chr22 involving the gene partners NUP214 and XKR3 [2]. The Hi-C data also showed clear evidence for a translocation between these loci; fine-mapping at the 50-kilobase scale revealed a likely breakpoint at chr9:133,050,000-133,100,000 and chr22:15,650,000-15,700,000. Further fine-mapping revealed that 14 reads (nearly all reads mapping to the 50-kilobase region) clustered tightly within a region at chr9:133,064,000-133,065,000 and chr22:15,680,000-15,681,000, suggesting that the breakpoint lies between the HindIII restriction sites in these regions.

Finally, we applied a similar fine-mapping procedure to identify breakpoint regions for a translocation t(3;10) that had been previously reported in a cytogenetic study [3]. This rearrangement has previously only been described based on its visual karyotypic appearance, and even the cytogenetic bands involved in this translocation have yet not been identified. The Hi-C data showed clear evidence for a translocation (Figure S1C; Figure S2B); we were able to fine-map the breakpoint, for the first time, to a region spanning chr3:48,150,000-48,200,000 and chr10:87,800,000-87,850,000. Hi-C read data was too sparse to perform further fine-mapping, but these regions overlap the CDC25A (cell cycle division 25A isoform A) transcript on chr3 and the GRID1 transcript on chr10.

Since CDC25A is an oncogene required for progression from G1 to S phase, we hypothesized that this translocation may result in aberrantly upregulated CDC25A. When we compared the expression of CDC25A in K562 cells [4] and an AML cell line [5], we found that CDC25A was approximately 8-fold higher in K562 cells ($P < 10^{-6}$, Student's *t* test). The significance and magnitude of this result was comparable to other dysregulated translocation partners in K562 cells (ABL1 and NUP214, Figure S2C-D). Translocation partners that supply regulatory elements (BCR, GRID1) and genes constitutively expressed in the myeloid lineage (MZF1, ELF4, and GAPDH) were not significantly upregulated. Interestingly, the breakpoint region on chr10 maps to an intronic region of the GRID1 transcript; within this 50-kilobase region, there are strong K562-specific H3K4Me1 histone marks and ChIP-seq derived

transcription factor binding sites that are not present in any other ENCODE cell type [6], suggesting that perhaps the fusion event either brings CDC25A under control of an existing enhancer, or creates a novel regulatory element that might drive CDC25A expression (Figure S2E). Our results suggest that the GRID1-CDC25A translocation may represent a novel functional fusion, although further characterization of this rearrangement will be required to define its exact functional role.

References

1. Ge H, Liu K, Juan T, Fang F, Newman M, Hoeck W. (2011) FusionMap: detecting fusion genes from next-generation sequencing data at base-pair resolution. Bioinformatics 27: 1922-1928.
2. Levin JZ, Berger MF, Adiconis X, Rogov P, Melnikov A, Fennell T, Nusbaum C, Garraway LA, Gnirke A. (2009) Targeted next-generation sequencing of a cancer transcriptome enhances detection of sequence variants and novel fusion transcripts. Genome Biol 10: R115.
3. Naumann S, Reutzel D, Speicher M, Decker HJ. (2001) Complete karyotype characterization of the K562 cell line by combined application of G-banding, multiplex-fluorescence in situ hybridization, fluorescence in situ hybridization, and comparative genomic hybridization. Leuk Res 25: 313-22.
4. Pellegrini M, Cheng JC, Voutila J, Judelson D, Taylor J, Nelson SF, Sakamoto KM. (2008) Expression profile of CREB knockdown in myeloid leukemia cells. BMC Cancer 8: 264.
5. Zhou J, Bi C, Chng W, Cheong L, Liu S, Mahara S, Tay K, Zeng Q, Li J, Guo K, Tan CPB, Yu H, Albert DH, Chen C. (2011) PRL-3, a metastasis associated tyrosine phosphatase, is involved in FLT3-ITD signaling and implicated in anti-AML therapy. PLoS One 6: e19798.
6. Kent WJ, Sugnet CW, Furey TS, Roskin KM, Pringle TH, Zahler AM, Haussler D. (2002) The human genome browser at UCSC. Genome Res 12: 996-1006.
