# Supplementary material for: Three-Dimensional Genome Architecture Influences Partner Selection for Chromosomal Translocations in Human Disease
Source: PLoS One. 2012 Sep 28;7(9):e44196. doi: 10.1371/journal.pone.0044196 (PMC3460994; doi:10.1371/journal.pone.0044196)
Supplement: Table S1 — Fine-mapping known K562 translocations. (PDF) [file pone.0044196.s007.pdf]

Table S1. Fine-mapping known K562 translocations.

Source	Reported K562 translocation	Evidence in Hi-C data?	Breakpoint region (hg18)	Annotated gene closest to predicted breakpoint
K562 Karyotype [1]	t(9;22)	Yes	chr9:132,550,000–132,600,000	chr9: ***ABL1***
			chr22:21,950,000–22,000,000	chr22: ***BCR***
	t(3;10)	Yes	chr3:48,150,000 – 48,200,000	chr3: ***CDC25A*** (cell cycle division 25A isoform A)
			chr10:87,800,000 – 87,850,000	chr10: ***GRID1***
	t(10;17)	Yes	chr10:42,200,000–42,250,000	chr10: *BC039000* (Homo sapiens cyclin Y-like 2)
			chr17:22,150,000–22,200,000	chr17: enhancer marks in K562
	t(9;17)	Yes	chr9:20,150,000–20,200,000	chr9: *MLLT3* (myeloid/lymphoid or mixed-lineage leukemia, translocated to 3)
			chr17:22,150,000–22,200,000	chr17: enhancer marks in K562
	t(5;6)	Yes	chr5:69,000,000–69,050,000	chr5: *GUSBP3*
			chr6:27,000,000–27,050,000	chr6: *GUSBP1*
	t(9;13)	No	-	-
	t(1;21)	No	-	-
	t(2;19)	No	-	-
	t(19;20)	No	-	-
	t(6;11)	No	-	-
	t(12;19)	No	-	-
K562 Next-Gen Sequencing [2, 3],*	t(9;22)_2	Yes	chr9: 133,050,000–133,100,000	chr9: ***NUP214***
			chr22: 15,650,000–15,700,000	chr22: ***XKR3***
	t(1;11)	No	-	-

*Targeted sequencing of 476 cancer-related gene cDNA transcripts only; did not detect non-coding breakpoints or translocations affecting other genes.

1. Naumann S, Reutzel D, Speicher M, Decker HJ. (2001) Complete karyotype characterization of the K562 cell line by combined application of G-banding, multiplex-fluorescence in situ hybridization, fluorescence in situ hybridization, and comparative genomic hybridization. Leuk Res 25: 313-22.

2. Ge H, Liu K, Juan T, Fang F, Newman M, Hoeck W. (2011) FusionMap: detecting fusion genes from next-generation sequencing data at base-pair resolution. Bioinformatics 27: 1922-1928.

3. Levin JZ, Berger MF, Adiconis X, Rogov P, Melnikov A, Fennell T, Nusbaum C, Garraway LA, Gnirke A. (2009) Targeted next-generation sequencing of a cancer transcriptome enhances detection of sequence variants and novel fusion transcripts. Genome Biol 10: R115.
