# Supplementary material for: Three-Dimensional Genome Architecture Influences Partner Selection for Chromosomal Translocations in Human Disease
Source: PLoS One. 2012 Sep 28;7(9):e44196. doi: 10.1371/journal.pone.0044196 (PMC3460994; doi:10.1371/journal.pone.0044196)
Supplement: Table S2 — Individual translocation-prone loci that significantly colocalize in normal nuclei. (PDF) [file pone.0044196.s008.pdf]

Table S3. Permutation results for NcoI Hi-C data.

Dataset	Total # Unique Translo cations	Translo -cations	Permut-ations	Permutation P-Value	T-test	Rank Sum	# of individually significant translocations
**Mitelman Database** (total)	577	0.15	0.00	<0.001	1.83E-29	4.84E-35	7
Blood	440	0.17	0.01	<0.001	4.69E-25	8.29E-30	4
Non-Blood	137	0.08	-0.04	<0.001	2.95E-06	3.05E-07	3
**Multiple myeloma**	89	0.11	-0.02	<0.001	4.48E-03	1.47E-02	2
**Prostate cancer**	89	0.00	-0.08	0.001	2.19E-03	3.41E-03	1
**Mendelian**	779	-0.02	-0.07	<0.001	1.09E-05	1.60E-04	2
