# Supplementary material for: Three-Dimensional Genome Architecture Influences Partner Selection for Chromosomal Translocations in Human Disease
Source: PLoS One. 2012 Sep 28;7(9):e44196. doi: 10.1371/journal.pone.0044196 (PMC3460994; doi:10.1371/journal.pone.0044196)
Supplement: Table S3 — Permutation results for NcoI Hi-C data. (PDF) [file pone.0044196.s009.pdf]

Table S2. Individual translocation-prone loci that significantly colocalize in normal nuclei.

Dataset	Karyotype	Permutation P-Value	Disease
Mitelman Database	t(12;19)(p13;p13)	<0.001	Acute lymphoblastic leukemia/lymphoblastic lymphoma
	t(12;21)(p13;q22)	<0.001	Acute lymphoblastic leukemia/lymphoblastic lymphoma
	t(3;12)(p21;p13)	<0.001	Acute lymphoblastic leukemia/lymphoblastic lymphoma
	t(7;12)(p15;p13)	<0.001	Acute myeloid leukemia, NOS
	t(3;19)(p21;p13)	<0.001	Acute lymphoblastic leukemia/lymphoblastic lymphoma
	tas(19;22)(q13;q13)	<0.001	Giant cell tumor of bone
	t(X;4)(p22;p12)	<0.001	Mantle cell lymphoma
Multiple Myeloma	t(4;14)(p16.3;q32.33)	<0.001	Multiple myeloma
	t(17;20)(p13.3;q11.22)	<0.001	Multiple myeloma
Prostate Cancer	none	-	-
Mendelian Disease	t(9;20)(q34;q11)mat	<0.001	Azoospermia/Oligozoospermia
