# Supplementary material for: Three-Dimensional Genome Architecture Influences Partner Selection for Chromosomal Translocations in Human Disease
Source: PLoS One. 2012 Sep 28;7(9):e44196. doi: 10.1371/journal.pone.0044196 (PMC3460994; doi:10.1371/journal.pone.0044196)
Supplement: Table S4 — Permutation results after controlling for chromatin compartment (HindIII). (PDF) [file pone.0044196.s010.pdf]

Table S4. Permutation results after controlling for chromatin compartment (HindIII).

		Mean Hi-Score		Permutation Method								
**Dataset**	**Total # Unique Translo cations**	**Translo -cations**	**Permut -ations**	**1**	**2**	**3**	**4**	**T-test**	**Rank Sum**	**# of individually significant translocations**	**% Genome Covered**	**% Interactions Covered***
**Mitelman Database** (total)	577	0.167	0.124	<0.001	0.002	<0.001	<0.001	7.8 E-04	1.2 E-03	6	79.7%	1.12%
**Multiple myeloma** (cytogenetic band mapping)	89	0.166	0.091	<0.001	0.003	0.007	0.001	9.7 E-02	1.7 E-01	5	44.8%	0.21%
**Prostate cancer** (cytogenetic band mapping)	89	0.071	0.014	<0.001	0.018	0.026	0.002	7.1 E-03	4.5 E-03	0	43.1%	0.19%
**Mendelian**	779	-0.047	-0.071	<0.001	0.004	<0.001	<0.001	1.6 E-02	2.1 E-01	6	91.5%	0.79%

*Percentage of inter-chromosomal 1-Mb bins that are covered by translocations.
